# Self‐Sustainable Wearable Textile Nano‐Energy Nano‐System (NENS) for Next‐Generation Healthcare Applications

**DOI:** 10.1002/advs.201901437

**Published:** 2019-10-24

**Authors:** Tianyiyi He, Hao Wang, Jiahui Wang, Xi Tian, Feng Wen, Qiongfeng Shi, John S. Ho, Chengkuo Lee

**Affiliations:** ^1^ Department of Electrical & Computer Engineering National University of Singapore 4 Engineering Drive 3 117576 Singapore Singapore; ^2^ National University of Singapore Suzhou Research Institute (NUSRI) Suzhou Industrial Park Suzhou 215123 China; ^3^ The N.1 Institute for Health National University of Singapore 28 Medical Drive, #05‐COR 117456 Singapore Singapore; ^4^ Centre for Intelligent Sensors and MEMS National University of Singapore 4 Engineering Drive 3 117576 Singapore Singapore; ^5^ Hybrid Integrated Flexible Electronic Systems (HIFES) 5 Engineering Drive 1 117608 Singapore Singapore

**Keywords:** healthcare, nanoenergy nano‐system (NENS), self‐sustainable, textiles, triboelectric

## Abstract

Wearable electronics presage a future in which healthcare monitoring and rehabilitation are enabled beyond the limitation of hospitals, and self‐powered sensors and energy generators are key prerequisites for a self‐sustainable wearable system. A triboelectric nanogenerator (TENG) based on textiles can be an optimal option for scavenging low‐frequency and irregular waste energy from body motions as a power source for self‐sustainable systems. However, the low output of most textile‐based TENGs (T‐TENGs) has hindered its way toward practical applications. In this work, a facile and universal strategy to enhance the triboelectric output is proposed by integration of a narrow‐gap TENG textile with a high‐voltage diode and a textile‐based switch. The closed‐loop current of the diode‐enhanced textile‐based TENG (D‐T‐TENG) can be increased by 25 times. The soft, flexible, and thin characteristics of the D‐T‐TENG enable a moderate output even as it is randomly scrunched. Furthermore, the enhanced current can directly stimulate rat muscle and nerve. In addition, the capability of the D‐T‐TENG as a practical power source for wearable sensors is demonstrated by powering Bluetooth sensors embedded to clothes for humidity and temperature sensing. Looking forward, the D‐T‐TENG renders an effective approach toward a self‐sustainable wearable textile nano‐energy nano‐system for next‐generation healthcare applications.

## Graphical abstract

**A universal strategy for enhancing the current and charging speed of textile‐based triboelectric nanogenerator (TENG)** toward a wearable textile Nano‐energy nano‐system for next‐generation healthcare applications is proposed. The soft, flexible, and thin TENG textile is capable of scavenging energy from various body motions effectively, and the enhanced current can directly stimulate both nerves and muscles.

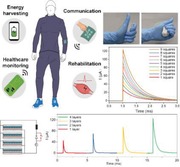

## 1 Introduction

As the world is marching into the era of the internet of things with the aid of remarkable progress on Fifth Generation, personal electronics and sensor networks have skyrocketed in the past few years.^1^, ^2^, ^3^, ^4^ Wearable and flexible electronics, owing to their promising future in a vast number of fields that are in a close relationship of human life, have drawn considerable attention from a broad spectrum of worldwide researchers.^5^, ^6^, ^7^, ^8^ To further improve the quality of human life, there is a need to provide quality care and services, especially in a nonclinical environment.^9^, ^10^ Wearable sensor networks have become a prominent technology allowing people to be monitored during their daily activities and help them by providing healthcare services such as medical monitoring,^11^ control of home appliances or various equipment,^12^, ^13^, ^14^ environmental monitoring,^15^ and communication in emergencies.^16^ Also, with the increase of the elderly population, age‐related diseases, including stroke and Parkinson's disease, are affecting more people. One of the direct consequences of these diseases could be the compromised movement capability, which requires further rehabilitation treatment, by stimulating either muscle or nerve, to maintain a certain level of life quality for the patients.^17^, ^18^, ^19^, ^20^

Remarkable progress on wearable electronics toward diversified applications such as human motions sensing,^21^, ^22^ healthcare monitoring,^23^, ^24^ and human–machine interfaces^25^ have been made in the past few years. The further development of wearable electronics is in high demand for widely distributed and mobile power sources, where most cases are still conventional batteries that cannot bypass the repetitive recharging processes.^26^ To this end, substantial efforts have been made for the development of environmentally friendly and sustainable energy harvesters that are capable of converting extensively existed mechanical energy into electrical energy based on the mechanism of piezoelectric,^27^, ^28^, ^29^, ^30^, ^31^ electromagnetic,^32^ and triboelectric.^33^, ^34^, ^35^, ^36^, ^37^, ^38^, ^39^ And the harvested energy could be used for powering diversified wearable sensors, or for direct muscle and nerve stimulation for rehabilitation, which enables a self‐sustainable wearable system for the next‐generation healthcare applications.

Textile, as a fundamental part of normal clothes, is well suitable for wearable applications due to its unique properties of light weight, soft nature, wearable comfortability, and wearable convenience.^40^, ^41^ The early generation of the electronic textiles (e‐textiles) adopts the strategy where the textile serves only as the substrate for the rigid or bulky electrical components to be integrated. To realize a seamless integration of the required functionalities and textiles, intrinsically flexible sensors made of textiles have emerged.^42^, ^43^ Benefiting from the particular advantages of various choices of materials, easy fabrication, simple working mechanism, and low cost, triboelectric nanogenerator (TENG) could be an optimal option for textile‐based energy harvesters and self‐powered sensors.^44^, ^45^, ^46^, ^47^, ^48^, ^49^, ^50^, ^51^, ^52^, ^53^, ^54^, ^55^, ^56^, ^57^, ^58^, ^59^, ^60^ However, the further advancement of the textile‐based TENGs (T‐TENGs) still faces challenges making its way to practical use due to the universal low output of them. To maintain a considerable output, the T‐TENG mostly follows the contact–separation design where one device is divided into two pieces that are placed separately on two different body parts, making it vulnerable to all kinds of damages and effects from the environment and limited to few working scenarios.^44^, ^45^, ^51^, ^54^, ^59^, ^60^ A simple and straightforward way to further improve the triboelectric output of the T‐TENG is the adoption of new material of high performance or surface modification, but it is still limited and increases the overall cost of the device.^54^ Besides, Pu et al. proposed a novel power‐textile working on the sliding mode of TENG to boost up the triboelectric output, yet the whole device is still separated by two parts and could be vulnerable to environmental variations.^59^ Through a sophisticated design on the knitted structure, Kwak et al. proposed a new way of output improvement for the T‐TENG, but with a sacrifice on the soft and thin nature of textile which is essential to provide a long‐term wear comfortability for users.^46^

To explore a broader prospect of TENGs, diversified mechanical switches to create instantaneous discharging are reported as an effective method to improve the triboelectric output. They are applied to various types of the TENGs, such as the common contact–separation designs,^61^, ^62^ the sliding design,^63^, ^64^, ^65^, ^66^ the rotating design,^67^ the multilayered TENG,^68^ TENG with two grounded electrodes,^69^ etc. However, this technique is limited to the rigid TENGs in which case highly synchronized motion patterns of the switch and the TENG are essential for the effective improvement. Recently, Xu et al. demonstrated using a high‐voltage diode to successfully control the charge flow of a rigid TENG without a sophisticatedly designed mechanical switch, which opens up a new possibility for achieving instantaneous discharging of TENGs.^70^

**Figure 1:**
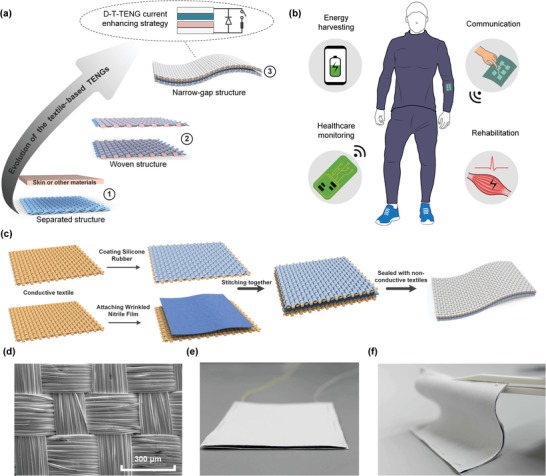
a) Road map showing the evolution of the T‐TENGs. b) Illustration of the wearable textile NENS for the next‐generation healthcare applications. c) Fabrication process of the T‐TENG. d) SEM image of the conductive textile. e,f) Images of the narrow‐gap and soft TENG textile on a table and curved by a tweezer.

Herein, for the first time, we integrate a T‐TENG with a high‐voltage diode and a mechanical switch as a facile and universal strategy to modulate the charge flow in the system to boost up the triboelectric output of flexible TENGs (**Figure** **1**a). A facile and easily controllable approach to create an instantaneous discharging with the narrow‐gap T‐TENG is proposed, as opposed to the sophisticatedly designed rigid TENGs. The closed‐loop current of the diode‐enhanced textile‐based TENG (D‐T‐TENG) has been improved by 25 times, and the charging speed over a capacitor has also been raised by four times. The D‐T‐TENG demonstrates a high prospect for the wearable textile nano‐energy nano‐system (NENS) for the next‐generation healthcare applications, which can provide diversified functionalities, including energy harvesting, healthcare monitoring, rehabilitation, and healthcare communication, as shown in Figure 1b.

## 2 Structure and Working Principle of the D‐T‐TENG

To perfectly fit the wearable TENG with regular apparels to scavenge waste energy from all kinds of human motion, a narrow‐gap T‐TENG in one piece is developed with a facile and low‐cost fabrication process as shown in Figure 1c. The device is composed of two layers, and both layers are conductive textiles covered with electrification materials. The positively triboelectric material is a thin and wrinkled film of nitrile, which is attached to the adhesive conductive textile loosely to create the irregular curvature on the contact surface. Another conductive textile is coated with silicone rubber and then stitched together with the nitrile‐coated textile face to face. Finally, the device is sealed with nonconductive textiles on the upper and bottom surface, with an overall thickness of around 1.2 mm. An SEM image of the conductive textile is provided in Figure 1d, and the photos of the T‐TENG placed on the table and curved by a tweezer are shown in Figure 1e,f, which demonstrate its flexible, soft, and thin characteristics that is well fitted with regular clothes for wearable applications.

**Figure 2:**
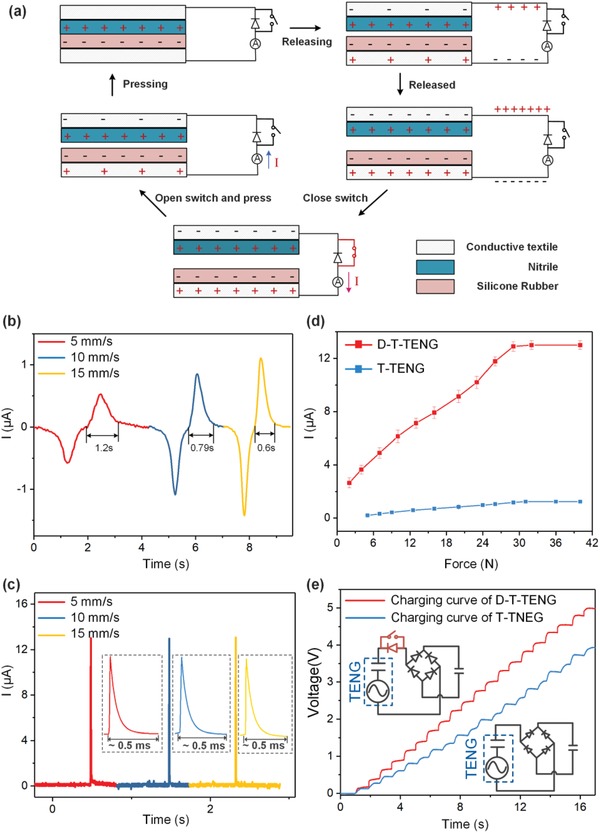
Basic characterization of the D‐T‐TENG. a) Working mechanism of the D‐T‐TENG. Current waveform of the b) T‐TENG and c) D‐T‐TENG under pressing/ releasing speed of 5, 10, and 15 mm s^−1^. d) Peak current of the D‐T‐TENG and T‐TENG as a function of applied forces at a constant pressing/releasing speed of 15 mm s^−1^. e) Charging curve of the D‐T‐TENG and T‐TENG on a 1 µF capacitor. The upper inset shows the circuit diagram of the D‐T‐TENG, and the bottom one is the circuit diagram for T‐TENG.

The working principle of the D‐T‐TENG is illustrated in **Figure** **2**a. As demonstrated by Xu et al., a high‐voltage diode connected in parallel with TENG can effectively accumulate charges on the two ends of the diodes to create a high open‐circuit voltage.^70^ Hence, a new approach to create an instantaneous discharging for TENG closed‐loop current enhancing is feasible with the integration of a high‐voltage diode for charge accumulation and a switch to short the diode for the instantaneous discharging in a controllable way. As shown in Figure 2a, the T‐TENG is connected to a high‐voltage diode and a switch in parallel, where the switch is only closed for a short time after the applied force is released from the TENG textile. The switch is only connected to the T‐TENG electrically, but not stacked together physically. The operation on the T‐TENG (i.e., pressing the TENG textile) and the operation on the switch (i.e., closing the switch) are individually controlled. As the TENG is pressed, the negative charges and positive charges are generated on the contact surfaces. While releasing, due to the electrostatic induction effect, negative charges tend to accumulate on the upper electrode to compensate the negative charges on nitrile and the positive inductive charges likewise accumulate on the bottom electrode. The charge transfer between the two electrodes in this process is prohibited by the high‐voltage diode. Hence opposite polarity charges of the same number of that on electrodes accumulate on the two ends of the diode to maintain the neutrality of the whole system. Upon closing the switch, the charges are immediately released in a very short period, generating a large spike current output. Therefore, the operation scheme of the D‐T‐TENG current enhancement strategy can be simplified into two steps happening in a time sequence: pressing TENG textile and releasing, closing the switch for a short time as demonstrated in Figure S1 in the Supporting Information. This instantaneous discharging process is similar to the discharging of a charged capacitor. Hence, the current profile would follow the shape of a typical discharging curve of a conventional capacitor, and it is only affected by the equivalent capacitance of TENG, the number of charges, and the external circuit.

For traditional TENG, the charge transfer processes between two electrodes happen gradually during the contacting and separating processes, which leads to a lower output peak current that is closely related with the contacting and releasing speed. To explicitly demonstrate the distinct characteristics of the D‐T‐TENG with the sole T‐TENG, a small piece of 3 cm × 3 cm of the narrow‐gap T‐TENG is adopted for the basic characterization. The short‐circuit current profiles of the sole T‐TENG when it is pressed by a fixed force of 40 N with varying speed from 5 to 15 mm s^−1^ are depicted in Figure 2b. It can be observed that the peak current increases with the pressing/releasing speed, and the pulse width gradually decreases with the increasing pressing/releasing speed. Similarly, the current profiles of the D‐T‐TENG on a load of 1 MΩ under different pressing/releasing speeds are measured and depicted in Figure 2c, where the insets show the enlarged current curves. The peak current of the D‐T‐TENG (about 13 µA) is 26 times higher than that of the T‐TENG (about 0.5 µA) when the contact/releasing speed is 5 mm s^−1^ and the pulse width is highly shortened to be around 0.5 ms. It can be explained that the accumulated charges of the D‐T‐TENG are instantaneously discharged as the switch is closed, which is the essential reason for the enhancing of the current. Apparently, the T‐TENG and D‐T‐TENG have a nearly equal number of inductive charges, while the D‐T‐TENG has a much higher discharging speed which is not limited by the mechanical contacting/releasing speed. Additionally, since the applied force is fixed at 40 N, the number of generated charges of the D‐T‐TENG is equivalent at different contacting/releasing speeds. Hence, it is observed that the current profile of the D‐T‐TENG stays unchanged because the equivalent capacitance of the D‐T‐TENG on the released state is unchangeable and the external circuit remains the same. Figure 2d shows the peak current values of the D‐T‐TENG and sole T‐TENG with variable applied force from 5 to 40 N, which indicates that both peak currents increase with the increment of force and saturate at around 30 N. The charging characteristics of the D‐T‐TENG and sole T‐TENG is also compared as shown in Figure 2e. A four‐layered T‐TENG with the same size of 3 cm × 3 cm is pressed by ten times at a constant frequency of 0.67 Hz and with the same force. The insets show the circuit connections of D‐T‐TENG and T‐TENG for charging a 1 µF capacitor. It can be charged up to around 4 V within pressing ten times with the sole T‐TENG, while this value is improved to 5 V with the D‐T‐TENG. This has shown the D‐T‐TENG not only can boost up the closed‐loop current but also be capable of enhancing the charging speed over a capacitor. The output characteristic of the D‐T‐TENG under different loads is provided in Figure S2 in the Supporting Information.

Up to now, various TENGs integrated with delicately designed mechanical switches for current enhancing have been reported, and the feasibility of current enhancement through instantaneous discharging is well proved. However, this approach can only be applied to a rigid design where the motion of the switch must be highly synchronized with the movable TENG, which requires a high cost on both design and fabrication of the switches. Differently, the D‐T‐TENG only needs to integrate a high‐voltage diode and a mechanical switch with the TENG for current enhancement, in which design the switch can be operated separately from TENG. And the integrated switch can work in various forms since there is no such requirement on the synchronization between the TENGs and the switches.

**Figure 3:**
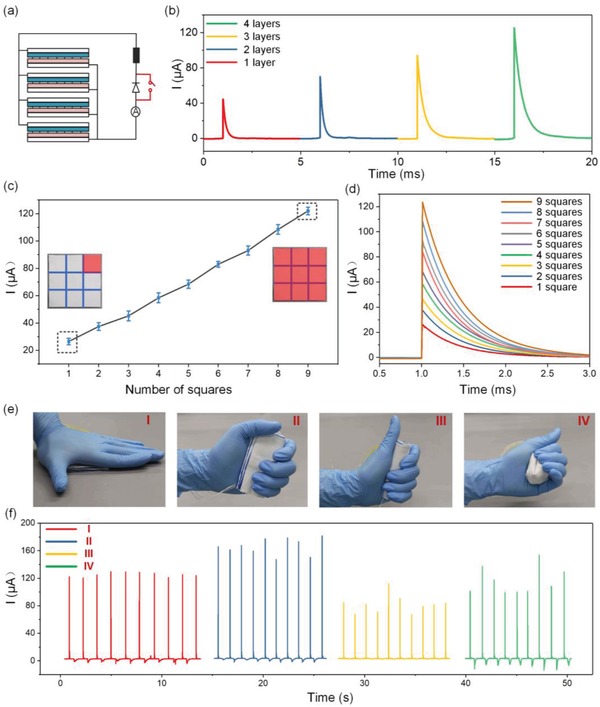
Current modulation of the D‐T‐TENG. a) Circuit diagram of the four‐layered D‐T‐TENG. b) Closed‐loop current on a 1 MΩ load of the D‐T‐TENG with different number of layers. c) Controllable peak current of the four‐layered D‐T‐TENG when pressed on different sizes of areas. d) Enlarged current profile of the D‐T‐TENG as the pressed area increases from one square to nine squares. e) Images of the four‐layered TENG textile being pressed normally (I), folded and pressed (II), rolled and pressed (III), random scrunched (IV). f) Corresponding current output under the four operation conditions.

To modulate the current output of the D‐T‐TENG, a TENG textile with a size of 8 cm × 8 cm is fabricated with four layers stacked together as shown in **Figure** **3**a. The current output of the D‐T‐TENG on a 1 MΩ load when it is pressed by hand with a force larger than 40 N is depicted in Figure 3b. From one layer to four layers, the peak current increases from around 44 to 125 µA, for the generated charges increases as the layers are connected in parallel. Meanwhile, it can be observed that the pulse width of the D‐T‐TENG also increases with the increment of the number of layers. The widened pulse width is due to the increased TENG equivalent capacitance when more layers are connected in parallel. On that account, the current profile including the peak current and the pulse width of the D‐T‐TENG can be modulated by controlling the device size and stacked layers. Besides, the peak current can be individually controlled by pressing the four‐layered device on areas of different sizes. Here the surface area of the device is equally divided into nine squares, and the peak current values as the device is pressed on different numbers of squares are depicted in Figure 3c. A good linear relationship between the peak current and the number of pressed squares can be seen, which is in accordance with the linearly increased contact areas. Figure 3d shows the corresponding enlarged current profiles of the four‐layered D‐T‐TENG, which indicates that the pulse width of the current stays almost the same when the pressed areas increases. Hence, a controllable current profile with a sole variable is achievable through varying the size of the pressed areas.

Beneficial from the soft, flexible, and thin nature of the TENG textile, it is able to work effectively under various conditions which include normal pressing, folded pressing, rolled pressing, and even random scrunching as shown in Figure 3e. The current of the D‐T‐TENG on a 1 MΩ load in the operating conditions as mentioned earlier is depicted in Figure 3f. It can be observed that the stability of the current profiles under normal pressing is superior to that of the other three motions. Besides, the peak current under folded pressing by hand is larger than others, which should be contributed by the larger contact area when it is folded and pressed. As the device is tightly rolled and pressed, the narrow gaps between the triboelectrification layers are further reduced, which gives rise to a much lower output than that of the other conditions. Even when the T‐TENG is randomly scrunched by hand, the peak current can still maintain a moderate value but with a large variation due to the inconsistency of the pressed areas. This high energy harvesting efficiency of the D‐T‐TENG under various working conditions opens up vast possibilities for scavenging waste energy from different body parts of distinct movement patterns.

## 3 Energy Harvesting from Various Body Parts

**Figure 4:**
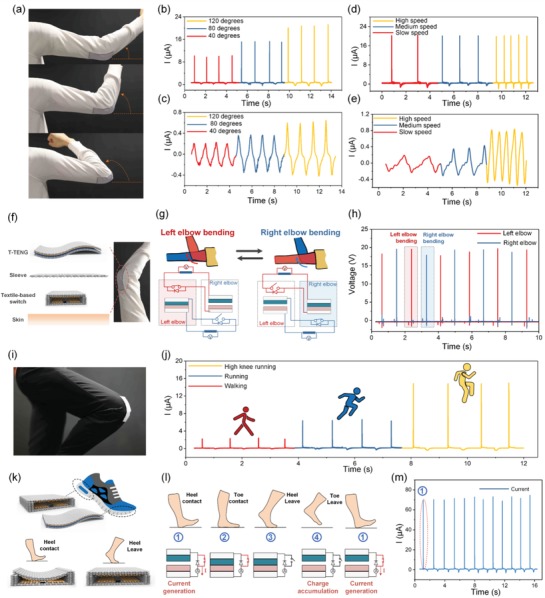
Energy harvesting from various body motions. a) Images of the elbow bending at around 40°, 80°, and 120°. Current of the b) D‐T‐TENG and c) T‐TENG as the elbow bending at different angles. d,e) Current of the d) D‐T‐TENG and e) T‐TENG when the elbow bends at 120° with ascending speeds. f) Exploded view of the functional sleeve embedded with T‐TENG on the outside and a textile‐based switch on the inside. g) Circuit diagram and working mechanism of the left‐elbow (red) and the right‐elbow (blue) D‐T‐TENG. h) Voltages waveform as the two elbows bend alternatively. i) Images of a TENG textile attached to the trousers on the knee location. j) Current of the D‐T‐TENG on knee as the user is walking, running, and doing high‐knee running. k) Images showing the locations of the TENG textile and the textile‐based switch (up), and the structure variation as the switch is pressed by heel (bottom). l) Working mechanism of the D‐T‐TENG on foot while walking. m) Current of the D‐T‐TENG while walking.

To demonstrate the energy harvesting capability of the D‐T‐TENG in real situations, it is integrated onto different body parts to scavenge energy from various body motions. First, a one‐layered 4 cm × 10 cm T‐TENG is fabricated and sewn on a hoodie jacket to harvest energy from elbow bending, as shown in **Figure** **4**a. For the D‐T‐TENG, after the charge accumulation period where the elbow bends to a certain degree and then returns to be straight, the switch should be closed for the instantaneous discharging. Here a textile‐based flexible mechanical switch is designed as a part of the D‐T‐TENG as shown in Figure S3 in the Supporting Information. Figure 4b,c shows the current on a 1 MΩ load of the D‐T‐TENG and the short‐circuit current of the T‐TENG when the elbow bends at different degrees while at the same speed. Similarly, the current of D‐T‐TENG and sole T‐TENG when the elbow bends at the highest degrees with an ascending speed is shown in Figure 4d,e. It can be observed that the peak currents of both T‐TENG and D‐T‐TENG increase with the growing bending degrees, which should be contributed by the higher contact force and larger contact area. When the elbow bends at the maximum degree with an ascending bending speed, the peak current of the D‐T‐TENG remains almost the same while the peak current of the T‐TENG increases with the growing speed because its discharging speed is limited by the mechanical contacting/releasing speed. On the one hand, this demonstrates the stability of the D‐T‐TENG over motion speed, showing that the D‐T‐TENG is well suited for scavenging energy from daily human activities due to the low‐frequency characteristic of most of the human motions.

As the switch is an indispensable part of the D‐T‐TENG for triggering the instantaneous discharging, it would be essential to discover the harmony between the human motion and the operation on the switches. Naturally, the human body is well symmetric, and a lot of movements are in an alternating mode where the two symmetric parts move one by one periodically. To make use of this alternating motion pattern of the elbow, here we assembled two textile TENG on the outside of both sleeves and two textile‐based switches on the inside of the sleeves at the same locations as illustrated in Figure 4f. The connection and working principles of the two D‐T‐TENGs are sketched in Figure 4g. Each D‐T‐TENG consists of a T‐TENG on one sleeve and a corresponding switch on the other sleeve. To be clear, the D‐T‐TENG with the switch on the left sleeve is marked as left‐elbow D‐T‐TENG which is sketched with the red lines on the upper side in Figure 4g, and the other one labeled right‐elbow D‐T‐TENG as depicted in blue lines. In this case, the charge accumulation process of the left‐elbow D‐T‐TENG is controlled by the bending of the right elbow, while the discharging process is triggered by the bending of the left elbow as highlighted in red in Figure 4g. Correspondingly, as the two elbows alternately bend one by one periodically, the charge accumulation and discharging processes of the left‐elbow D‐T‐TENG and the right‐elbow D‐T‐TENG are reversely staggered. The voltage outputs across a 1 MΩ load of the two D‐T‐TENGs under this alternating elbow bending movement at 120° are measured and depicted in Figure 4h. A well alternating pattern of the two outputs can be observed, where each peak represents the bending of the left or right elbow, as highlighted in red or blue in Figure 4h.

Similar to the elbow, the knee could also be an integration location of the D‐T‐TENG for mechanical energy harvesting. The same size device is attached to the outside of the jeans as shown in Figure 4i, and the current of the D‐T‐TENG on a 1 MΩ load while walking, regular running, and high knee running are measured (Figure 4j). Due to the curvature of the knee, the T‐TENG is still slightly pressed even when the leg is fully relaxed, and the maximum bending degree of the knee is only around 90°. Both characteristics contribute to a lower peak current compared to that of the D‐T‐TENG on the elbow. Besides, the bending of the knees is quite minor under normal walking and running motions if not bending it on purpose. Hence the peak current in these two conditions is much smaller.

As characterized previously, the current output of the D‐T‐TENG only increases with the applied force and shows no relevance to the mechanical contacting/releasing speed. Foot, as a body part that bears the highest force on a daily basis, is an ideal location for energy harvesting. It is known that the movements of the heel and the toe are likewise in an alternating pattern that could be used for the integration of the D‐T‐TENG naturally. In this case, a six‐layered T‐TENG with a size of 4 cm × 6 cm is fabricated to fit the size of foot and attached inside of a shoe on the front side. A textile switch is put inside of the shoe on the rear end as demonstrated in Figure 4k. When the heel is contacting the ground, the textile‐based switch is pressed by heel which leads the upper conductive textile to be pressed down to contact with the bottom conductive textiles (Figure 4k‐ii), and hence the diode is shorted for the instantaneous discharging. The working principle of the D‐T‐TENG inside of a shoe to scavenge energy from foot motions is depicted in Figure 4l. Starting from phase 2, the T‐TENG is pressed by the bodyweight as the toe contacts the ground after heel contact. Then heel leaves first followed by toe leaving (phase 3 and 4), which corresponds to the charge accumulation process of the D‐T‐TENG. Next, the heel contacts the ground and closes the switch to trigger the instantaneous discharging at the end of one cycle as marked in phase one. While walking or running, the four phases happen in sequence repeatedly as the foot marches forward step by step. The output current of the D‐T‐TENG on a 1 MΩ load while walking at a low frequency of around 0.7 Hz is sketched in Figure 4m, where each peak corresponds to the heel contact motion in every cycle.

## 4 Rehabilitation Application

Energy harvested from body motions can directly stimulate biological tissues without any rectification. For peripheral nerve stimulation, Zhang et al. first demonstrated the electrical activation of the frog sciatic nerve directly powered by a TENG.^71^ Then, Lee et al. further investigated the high electrical activation efficiency as well as the selectivity of the rat sciatic nerve powered by TENGs.^17^, ^19^ More recently, Wang et al. first reported direct muscle stimulation with output from TENGs.^18^ However, the current available TENGs, especially flexible TENGs, suffer from low current output, which is only µA‐level. Hence the device size used for the direct muscle stimulation was large to obtain enough current, and it is not suitable for actual wearable applications. For nerve stimulation, a µA‐level current is enough to penetrate the nerve tissue and induce force output. But muscle typically requires much larger current to be stimulated.^72^ As a result, the µA‐level current from TENGs can only activate some motoneurons in the muscle. Here, we want to test if the enhanced current output from D‐T‐TENGs help to increase the force output during muscle stimulation, as well as how the increased current output affects force output during muscle and nerve stimulation.

**Figure 5:**
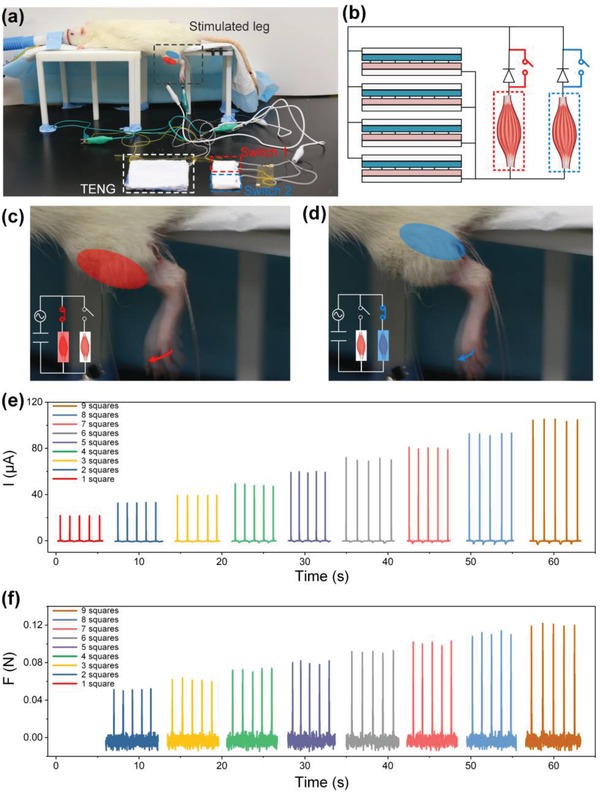
Direct muscle stimulation with the D‐T‐TENG. a) Images of the testing set up for the rat tibialis anterior muscle and gastrocnemius muscle stimulation. b) Circuit diagram of selective muscle stimulation. c) Image of the rat leg motion when the tibialis anterior muscle is stimulated. d) Image of the rat leg motion when the tibialis gastrocnemius muscle is stimulated. e) Current profile over a 1 MΩ load of the D‐T‐TENG when different number of squares are pressed. f) The corresponding force profile of the rat leg when it is induced to move forward by pressing different number of squares of the D‐T‐TENG.

To test the in vivo muscle stimulation capability of the D‐T‐TENG, we chose the tibialis anterior muscle and gastrocnemius muscle for demonstration, which controls the forward kicking and backward kicking of the rat leg respectively (**Figure** **5**a). In each muscle, we implanted a pair of stainless‐steel wire electrodes to form a closed loop to allow current flow from the D‐T‐TENG to the muscles. Each pair of the stainless‐steel wire electrodes was connected to a separate switch, and these two switches share the same TENG, which means we could manually change to deliver current flow to a muscle by changing the pressed switch (Figure 5b). During the testing, the rat was under anesthesia and was put on a stand with the leg hanging freely, to allow observation of kicking forward by stimulating the tibialis anterior muscle as shown in Figure 5c or kicking backward by stimulating the gastrocnemius muscle as shown in Figure 5d (Video S1, Supporting Information).

As shown in Figure 3c, during benchtop testing, we achieved linear current output by changing the activation area. Here, we also wanted to test how the linear current output would affect muscle stimulation force output. For demonstration, we chose to stimulate the tibialis anterior muscle. The leg was tied to a force gauge through a wire, so that force output was recorded and digitized by the force gauge for further quantized analysis (Figure S4, Supporting Information). Figure 5e shows the linear correlation between the current output and the activation area, which was tested during the in vivo measurement, to provide an on‐site calibration of the D‐T‐TENG output characterization. Figure 5f shows the force output measurement result of using the same D‐T‐TENG to stimulate the tibialis anterior muscle. It turns out that force output increases linearly with the stimulation current. This is desirable for prosthesis control, as the linear correlation simplifies the stimulation current control. With such concise linear correlation, we can easily calculate the required activation area to achieve a certain desired force output. This experiment proofed the capability of linear force control by changing the activation area of D‐T‐TENG and opens an opportunity for future prosthesis control.

**Figure 6:**
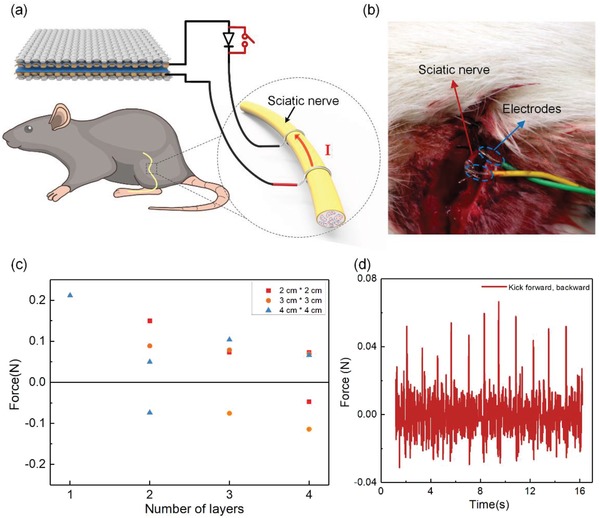
Direct nerve stimulation with the D‐T‐TENG. a) Image showing the testing set up for rat sciatic nerve stimulation. b) Enlarged photo of the sciatic nerve connected to the stimulation electrodes. c) Force distribution of the leg motions when it is stimulated by D‐T‐TENG with different sizes and layers. d) Recorded force profile when the sciatic nerve is stimulated by a four‐layered D‐T‐TENG with a size of 2 cm × 2 cm.

Furthermore, we also tested the stimulation capability of the D‐T‐TENG on the sciatic nerve of the rat. Nerve stimulation is different from muscle stimulation, as the nerve has a much smaller size as compared to the muscles, and as a result, the electric field may spread out to affect the whole nerve more easily. Here, we chose to demonstrate the stimulation capability using D‐T‐TENG on the sciatic nerve on the rat leg, as the sciatic nerve is a large peripheral nerve and easy to operate on. The implantation is shown in **Figure** **6**a,b), where we tied two stainless steel wire electrodes to the sciatic nerve to form a closed loop with the D‐T‐TENG. To the distal end, the sciatic nerve branches into three smaller nerves, namely, the common peroneal nerve, the tibial nerve, and the sural nerve, to innervate different muscles. Here, since we were delivering electrical stimulation to the main sciatic nerve, the electric field would spread out to affect the three branches. We would like to study how the effect varies with the stimulation current, so we used D‐T‐TENG of different sizes, with layers ranging from one to four layers and size ranging from 1 cm × 1 cm to 4 cm × 4 cm. The quantized force output is summarized in Figure 6c, with the raw data of the recorded force output shown in Figure 6d. For the largest 4 cm × 4 cm D‐T‐TENG stimulation, even one single layer induced force output, while the smaller D‐T‐TENGs required more layers to successfully stimulate the sciatic nerve. An interesting observation is that stimulation with certain device size and certain layers not only induced forward‐kicking force but also induced visible backward‐kicking force, which were characterized as the positive and negative force in Figure 6d, respectively. Here, we suspect that electric field spread out to activate different areas of the sciatic nerve with the increasing stimulation current. When current amplitude reached a certain level, the nerve branch controlling backward‐kicking gastrocnemius muscle also contributed force, which would be measured as the negative force.

As compared to the conventional triboelectric device for direct muscle stimulation, where the harvested current is low and the electrode needs to be specially designed for high stimulation efficiency,^18^ our D‐T‐TENG enables high‐amplitude current output for optimized stimulation results with simple electrodes. For the consideration of simple therapeutic implementation and future translation to human clinical applications, our D‐T‐TENG with large current output is preferred. In addition, made of soft and comfortable fabric material, the D‐T‐TENGs would show their capability in wide rehabilitation applications, not limited to muscle and nerve electrical stimulation for movement facilitation. Moreover, if the intramuscular electrode and the corresponding stimulation strategies are combined with recently developed implantable energy‐harvesting devices, an implantable and self‐powered rehabilitation system could be potentially realized in near future.^73^, ^74^, ^75^

## 5 Healthcare Communication

**Figure 7:**
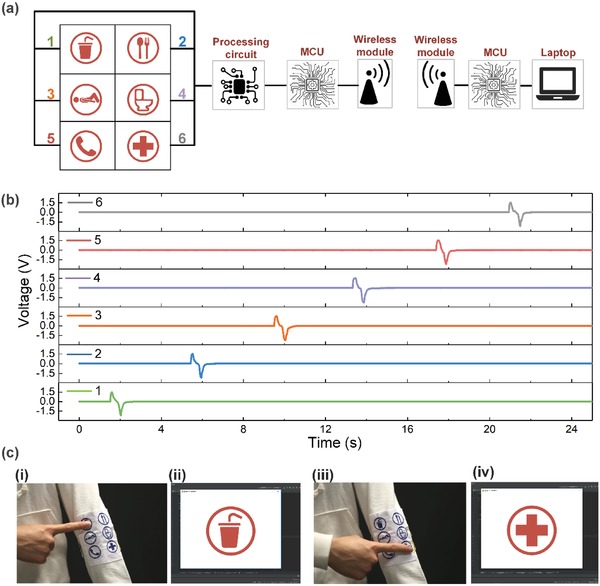
Wearable wireless communication board. a) Circuit connection of the wireless communication board system. b) Voltages signals collected from the MCU as the six pixels are pressed one by one. c) Photos of the textile‐based communication board integrated on an actual garment when it is pressed on the first (i) and last (iii) pixel, and the corresponding icons displayed on the screen (ii and iv).

Apart from directly used for rehabilitation applications, the T‐TENG itself is also capable of providing a sensing signal with simple functionalities in home‐care or hospital‐care services. For the elderly or patients with limited mobility, nurses depend upon their communicative skills to be able to understand and essential needs to be met. On a daily basis, the patients may need physical care on various occasions, which requires task‐related communication. To satisfy the need of the patients or the elderly for support, recognition, and understanding, a textile‐based wireless communication board is fabricated with six pixels, where each pixel contains a T‐TENG square with a size of 2 cm × 2 cm. Unlike a keyboard where the user has to type the whole word at one time, each pixel of the textile‐based communication board can be predefined based on the most frequent and essential activities required from the patients or the elderly. Here, a graphic icon sewn with threads on top of each pixel is able to provide a user‐friendly interface for the patients, especially the elderly, to quickly pick up the simple and obvious operation scheme. Here the six pixels are defined as drink, food, lying down/sitting up, bathroom, emergency contact, and calling the hospital, as shown in **Figure** **7**a.

The circuit connection for the whole system is illustrated in Figure 7a, where each T‐TENG is connected to a microcontroller unit (MCU) through a preprocessing circuit for noise elimination. The analog outputs collected from the T‐TENGs are transferred into digital signals to be transmitted out through a wireless module. Then the wireless signals are received with another wireless module and sent to the computer through the MCU as a demonstration. The input voltages of the MCU connected to the textile board when the six pixels are pressed one by one are sketched in Figure 7b. Each simple pressing generates a peak voltage of around 1.5 V. To demonstrate the seamless integration of the textile‐based wireless communication board with the common apparel, it is attached to the outside of the sleeve of a jacket. As shown in Figure 7c–i, when the index finger presses the drink icon, a corresponding picture of the drink icon is shown on the screen of the computer. Similarly, as the finger moves to the sixth pixel and presses down, the hospital icon appears demonstrating effective and accurate communication (Video S2, Supporting Information). The functionality of the communication board can be further broadened through incorporating more pixels that can be customized based on the practical situation of each patient, which is highly beneficial for the establishment of an efficient means of communication with medical professionals during hospital stays or at home.

## 6 Energy Harvesting for Healthcare Monitoring

**Figure 8:**
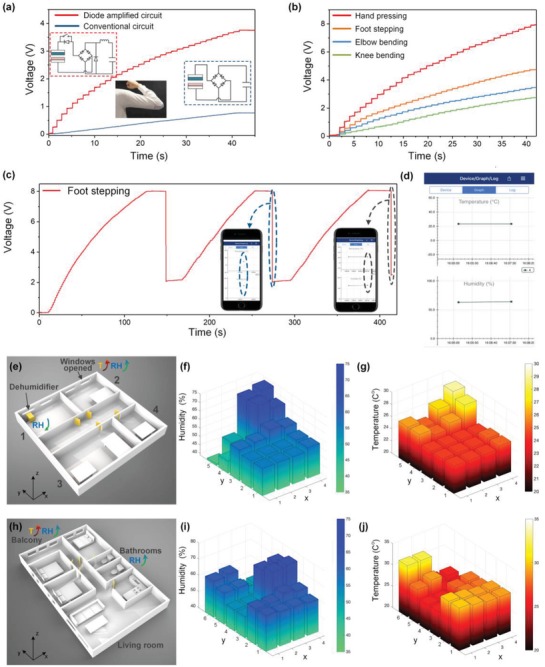
D‐T‐TENG powered Bluetooth sensing. a) Charging curves on the 27 µF capacitor of the D‐T‐TENG and T‐TENG as the elbow bends 120°. The insets show the circuit diagram for the D‐T‐TENG (left) and for the sole T‐TENG (right). b) Charging curves with the D‐T‐TENGs from different body parts. c) Charging and discharging curve with the D‐T‐TENG on foot, where each voltage drop represents a discharging to the Bluetooth module. d) Enlarged screenshot on a smartphone showing the collected information from the Bluetooth module. e) 3D layout of a lab environment. f,g) Humidity and temperature distribution of the lab environment with 20 sampling points. h) 3D layout of an apartment. i,j) Humidity and temperature distribution of the apartment with 24 sampling points.

Besides directly using the enhanced output current of the D‐T‐TENG for muscle or nerve stimulation, the functionality of the D‐T‐TENG could be further broadened by storing the scavenged energy to power healthcare monitoring sensors. Here the charging circuit of the D‐T‐TENG is optimized with the incorporation of a classical bulk converter to further step up the charging speed. For harvesting energy from actual body motions, the pressing and releasing of the T‐TENG mostly happen gradually, in which case the charging speed with the conventional charging circuit could be much smaller compared to that of the ideal situation where the force is evenly distributed over the pressing/releasing processes. To compare the charging capability of the D‐T‐TENG and sole T‐TENG when harvesting energy from practical body motions, a three‐layered 4 cm × 12 cm T‐TENG is attached to the elbow to charge a 27 µF capacitor while bending at a maximum bending degree with a frequency of around 0.67 Hz (**Figure** **8**a). The circuit connection for the D‐T‐TENG is illustrated as the inset highlighted in red square, and the sole T‐TENG is connected to the capacitor with the conventional circuit shown in the blue highlighted inset. After 35 times of bending at the largest degree, the 27 µF capacitor is charged up to around 3.8 V by the D‐T‐TENG, which is almost five times of the charged‐up voltage by sole T‐TENG. This has further indicated the superiority of the D‐T‐TENG over the sole T‐TENG in charging speed, especially under practical human motions. Besides, the energy harvesting capabilities of different human motions are also evaluated as shown in Figure 8b. To have a fair comparison, for the hand pressing and foot stepping, the device is a six‐layered 4 cm × 6 cm T‐TENG, while a three‐layered 4 cm × 12 cm T‐TENG is adopted for elbow bending and knee bending tests so that the overall contact areas are in the same size. With the optimized circuit connection, it can be observed that hand pressing gives the largest charging speed, and followed by the foot pressing, elbow bending, and knee bending in the descending order. The highest charge accumulation speed was measured to be 5.68 µC s^−1^ (calculated by Δ*Q* = *C*Δ*V*), which is much higher than what has been reported in the previous reported T‐TENGs (Table S1, Supporting Information). The lower charging speed on foot stepping condition compared to hand pressing may be contributed by the curved surface of the forefoot leading to an insufficient contact and the partial releasing under actual walking. For the elbow bending and knee bending, the smaller charging speed results from the less applied pressure over the T‐TENG during the entire bending motions compared to direct pressing and stepping.

The effect of temperature and humidity on the perception of odor intensity and air quality has been investigated intensively. It has been proved that both too low or high relative humidity may cause some physical discomfort which could lead to some severe diseases in the long term. It is also reported that extremely high or low temperature has been associated with deaths among the elderly and also young children, which are both large susceptible populations. Human's healthcare and wellness assessment in living and medical environments may benefit from continuous and reliable monitoring of critical parameters such as the temperature and humidity.^76^ To demonstrate the feasibility of the D‐T‐TENG as a possible power source for healthcare monitoring sensors in daily life, here a low‐power Bluetooth module incorporated with a humidity sensor and a temperature sensor is powered by the 27 µF capacitor charged with the D‐T‐TENG on foot. After charged up to 8 V, the 27 µF capacitor is connected to the Bluetooth module as shown in Figure S5 in the Supporting Information. It is able to work for 1 s after each discharging, after which the voltage on the capacitor drops to 2 V. Each time the Bluetooth module will collect the environmental humidity and temperature data from the embedded sensors and send it out to the smartphone, where the users can track both parameters. The charging and discharging curve in three cycles with the D‐T‐TENG on foot for the Bluetooth module powering is sketched in Figure 8c. Within 80 times of stepping in normal walking, the 27 µF capacitor can be charged up to 8 V for powering. The first discharging to the Bluetooth module works as a triggering at which state the Bluetooth module emits no signal. As what can be seen in Figure 8c, each discharging leads to the voltage drop on the 27 µF capacitor from 8 to 2 V; hence the charging time for the subsequent cycles is further shortened. Figure 8d shows the enlarged screenshot of the smartphone displaying the collected signals from the Bluetooth module after discharging for two times. A video showing the D‐T‐TENG powering the Bluetooth module by foot stepping is provided in Video S3 in the Supporting Information.

Here we used the Bluetooth module to map the temperature and humidity distribution of two typical environments. Figure 8e shows a 3D model of a lab environment, which contains four rooms with central air conditioner coverage and a corridor. Total 20 sampling points are evenly distributed over the whole area, and the corresponding temperature distribution and humidity distribution is sketched in Figure 8f,g. Due to the existing central air conditioner and the absence of windows in room 3 and room 4, the temperature and humidity in this area are quite stable (Figure 8f,g). A steep increase of both humidity and temperature in room 2 region is observed due to the opened windows where the hot and wet air leaks in. A dehumidifier is placed in the corner in room 1, and correspondingly a low humidity of around 40% is observed around point (1, 5). Besides, a slight increase in temperature caused by the hot air blown by the dehumidifier is also visible from the graph. Figure 8h demonstrates a 3D layout of a typical living environment, which contains three bedrooms with air conditioners embedded. There are 24 sampling points in total, which are evenly distributed over the whole area. It can be observed that the humidity and temperature inside all three bedrooms are stable and smaller than that of the outside environment as shown in Figure 8I,j. And the highest temperature distribution over the balcony region is consistent with its location where receives the highest Sun exposure at that time. A sudden increase in the humidity level is observed inside the bathrooms, which should be contributed by the insufficient air circulation caused by the lack of windows. This has revealed the imperfect designing of the bathrooms that can have far‐reaching effects on the longevity of the bathroom area and its immediate surrounding rooms, as well as adverse effects on the health of the people using the bathrooms.

As what has been demonstrated, the D‐T‐TENG can function as a wearable power source for wireless humidity and temperature sensing, which could help the users collect and track the environmental temperature and humidity levels while traveling between places so as to avoid associated health effects in the long term. Besides, with the incorporation of more low‐power sensors with different functionalities such human vital signs monitoring, smart apparel integrated with D‐T‐TENG and various sensors for a comprehensive healthcare monitoring is feasible soon.

## 7 Conclusion

In summary, a narrow‐gap T‐TENG is fabricated with a facile and low‐cost fabrication process, and a universal strategy for improving the triboelectric output of the flexible especially T‐TENGs is proposed and investigated. With the integration of a high‐voltage diode and a mechanical switch, the diode‐amplified T‐TENG (D‐T‐TENG) is able to generate a high closed‐loop current which is 25 times higher than that of the sole T‐TENG, and the speed of charging capacitors is also increased by around four times. Due to the soft and thin characteristics, the D‐T‐TENG can work under various conditions while still maintaining a moderate output. Besides, the enhanced output current is high enough for both muscle and nerve stimulation, which can be easily modulated to realize a controlled muscle stimulation. The TENG textile is also used to build a wearable communication board to provide a simple yet effective communication approach between the nurses and the patients, convalescent, or the elderly to facilitate the care quality in daily life. In addition, the D‐T‐TENG is used to harvest energy from normal walking to power a Bluetooth module embedded to clothes for environment humidity and temperature sensing. With the improvement on the charging speed, the Bluetooth module can work for 1 s within only 70 times of stepping of one foot, at which time the humidity and temperature value is sent to the smartphone for continuous tracking. Looking forward, diversified sensors can be embedded to the textile for the construction of a wearable sensor network on common clothes, and self‐powered sensors and energy harvesters are key prerequisites to realize a self‐sustainable system. Therefore, the D‐T‐TENG, which has opened up countless possibilities for distributed power sources over the whole body, paves the way to the wearable textile NENS for the next‐generation healthcare applications.

## 8 Experimental Section

*Fabrication of the Textile‐Based TENG*: The conductive textile was made of metallized fabric (polyester Cu) coated with an adhesive. The device was composed with two layers, both layers were conductive textiles covered with electrification materials. The triboelectric positive material was a thin film of nitrile, which was simply attached to the adhesive conductive textile loosely to create the random curvature on the contact surface. The negatively triboelectric layer here was a silicone rubber thin film. After dispensing required amounts of Parts A and B of the EcoFlex 00‐30 into a mixing container (1A:1B by volume or weight), the blend was mixed thoroughly for 3 min, and then the mixed solution was pasted onto the conductive textile surface followed by a 20 min baking at 70 °C for curing. The two layers were stitched together on the edges by cotton threads together with nonconductive textiles on the upper and bottom as sealing.

*In Vivo Electrode Implantation*: All experiments were conducted according to protocols approved by the Institutional Animal Care and Use Committee at the National University of Singapore. Six Sprague‐Dawley rats (around 450 g) were used for the acute experiments. Anesthesia (Aerrane, Baxter Healthcare Corp., USA) was induced with isoflurane. Carprofen (Rimadyl, Zoetis, Inc., USA) was injected for pain relief before surgery. After the rat was anesthetized, fur on the leg was gently removed by a shaver. Then, the skin was disinfected with 70% ethanol wipes, and an incision was made with a surgical blade to expose the tibialis anterior muscle (TA muscle). The stainless steel wires were sutured into the TA muscle belly.

*Force Data Collection and Analysis*: The anesthetized rat was fixed on a stand, and the ankle of left leg was connected to a dual‐range force sensor (Vernier, USA). This force sensor was connected to a laptop through a data acquisition (DAQ). LabView (National Instruments, USA) was used for on‐site result visualization during the measurements. After the measurements, MATLAB was used for data analysis.

*Study Participation*: Prior to participation in the experiments, informed consent was obtained from the volunteer in all experiments.

## Conflict of Interest

The authors declare no conflict of interest.

## Supporting information

Supplementary

Supplemental Video 1

Supplemental Video 1

Supplemental Video 1
