# Supplementary material for: Self‐Sustainable Wearable Textile Nano‐Energy Nano‐System (NENS) for Next‐Generation Healthcare Applications
Source: Adv Sci (Weinh). 2019 Oct 24;6(24):1901437. doi: 10.1002/advs.201901437 (PMC6918113; doi:10.1002/advs.201901437)
Supplement: Supplementary file 1 — Supplementary [file ADVS-6-1901437-s001.pdf]

Copyright WILEY-VCH Verlag GmbH & Co. KGaA, 69469 Weinheim, Germany, 2019.

ADVANCED SCIENCE
Open Access

Supporting Information

for *Adv. Sci.,* DOI: 10.1002/advs.201901437

Self-Sustainable Wearable Textile Nano-Energy Nano-System (NENS) for Next-Generation Healthcare Applications

*Tianyiyi He, Hao Wang, Jiahui Wang, Xi Tian, Feng Wen, Qiongfeng Shi, John S. Ho, and Chengkuo Lee**

Copyright WILEY-VCH Verlag GmbH & Co. KGaA, 69469 Weinheim, Germany, 2018.

Supporting Information

Self-Sustainable Wearable Textile Nano-Energy Nano-System (NENS) for The Next-generation Healthcare Applications

*Tianyiyi He, Hao Wang, Jiahui Wang, Xi Tian, Feng Wen, Qiongfeng Shi, John S. Ho, and Chengkuo Lee**

Table S1. Charging speed comparison with the reported textile-based TENGs.

Device configuration [a)]	Motion speed or frequency	Charging speed per volume [nC s^{-1} cm^{-3}] [b)]	Ref
Two pieces	2 Hz	12.35	[44]
Two pieces	75 cm/s	41.3	[59]
Two pieces	10 cm/s	2.4	[60]
Two pieces	2 Hz	35	[52]
Single fiber	1 Hz	76	[47]
Two pieces	2 Hz	25.5	[45]
Two pieces	2 Hz	61	[51]
Two pieces	3 Hz	40	[50]
Two pieces	4 Hz	132	[54]
Two pieces	1 Hz	75	[53]
One piece	1 Hz	1.7	[57]
One piece	0.67 Hz	263	Our work

[a)] The device configuration is simply defined in two categories. “Two pieces” represents the triboelectric positive part and negative part are separated attached to different body parts. “One piece” means the triboelectric positive and negative parts are integrated together in one piece; [b)] The charging speed per volume is estimated by $\Delta Q/ (t \times V) = C \times U/ (t \times V)$, where the accumulated charge (ΔQ) equals to the product of capacitor size (C) and the charged voltage (U). Then it is divided by the charging time and device size to get the charging speed per volume.

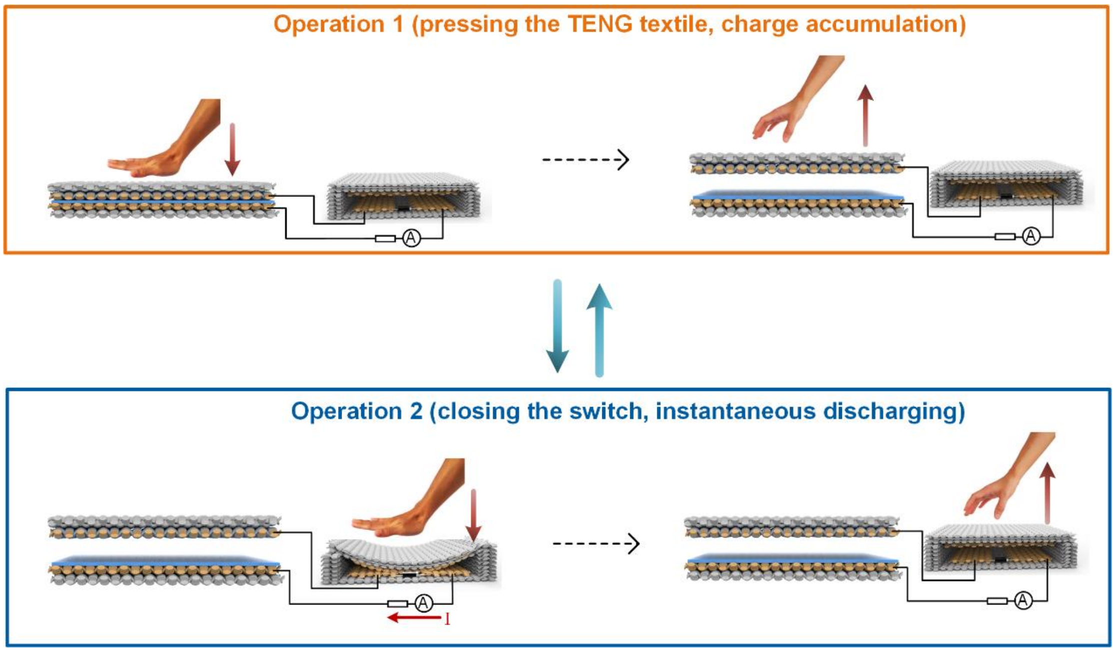

Figure S1. Illustration of the operation scheme of the D-T-TENG to generate a pulsed output. Operation 1 is to press the TENG textile and then release the force, in which process the charges are accumulated at the two ends of the diode. Operation 2 is to close the switch for a short time for the instantaneous discharging.

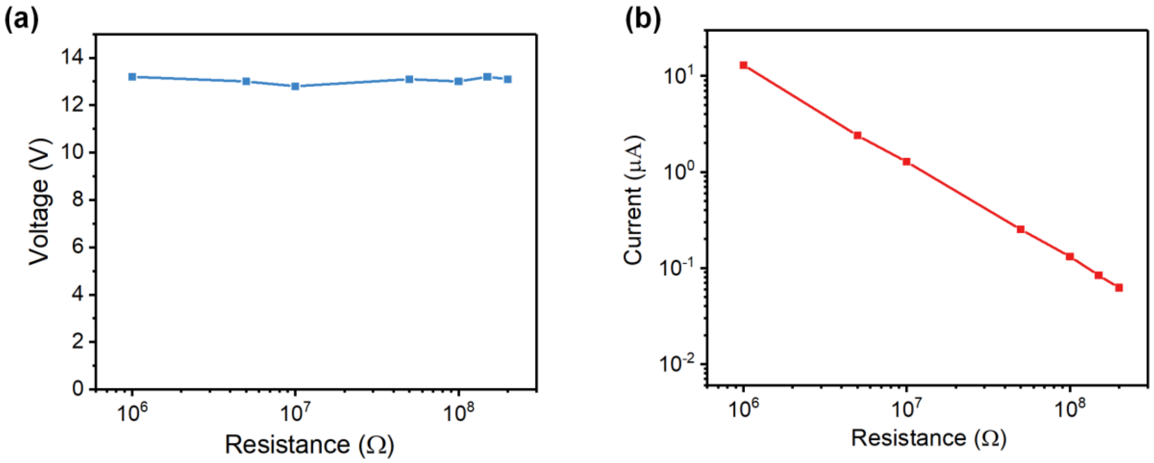

Figure S2. The dependence of (a) peak voltage, and (b) peak current of the D-T-TENG (3 cm × 3 cm, 1 layer) on load resistance. Due to the unique instantaneous discharging which is similar to the discharging of the charged capacitor, the peak voltage is unaffected by the load resistance but only related to the number of accumulated charges. Hence it is observed that the peak voltage (V_{max}) stays the same as the load increases. On that account, the peak current (I_{max}) passing through the load is a reverse function of the load because of $I_{max} = V_{max}/R$.

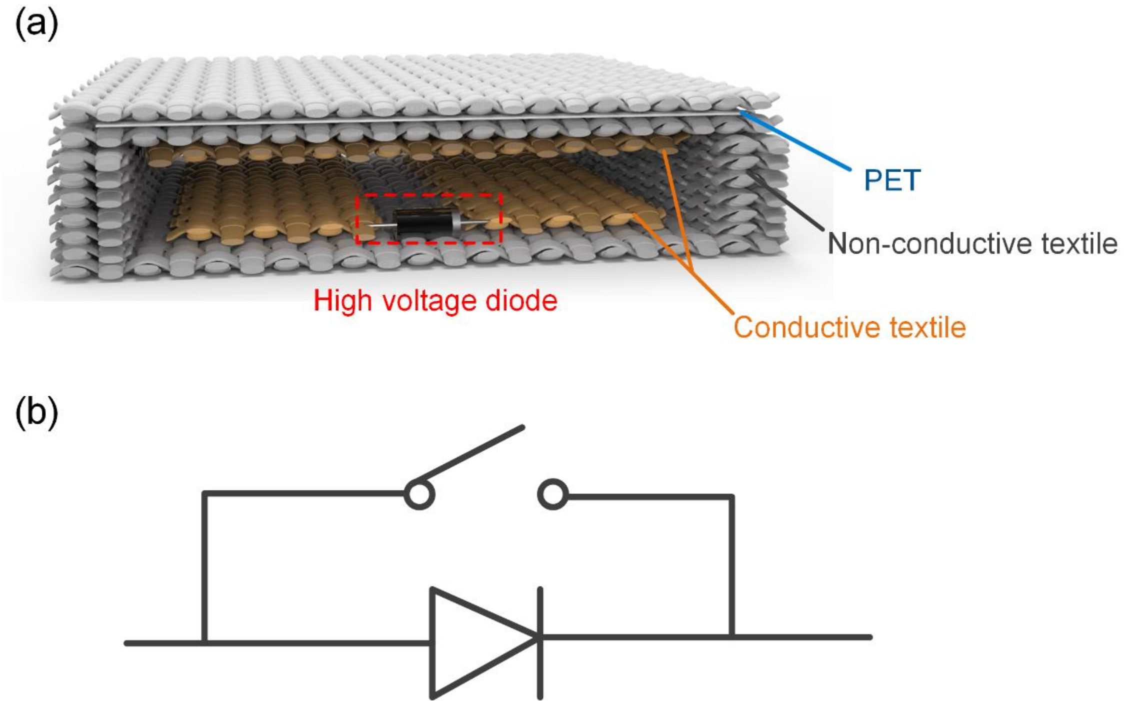

Figure S3. Device configuration of the textile-based switch. a) 3D structure of the textile-based switch. Here a thin layer of PET was inserted into the top textile layer to improve the long-term stability and robustness of the switch. b) A corresponding simplified circuit diagram of the textile-based switch.

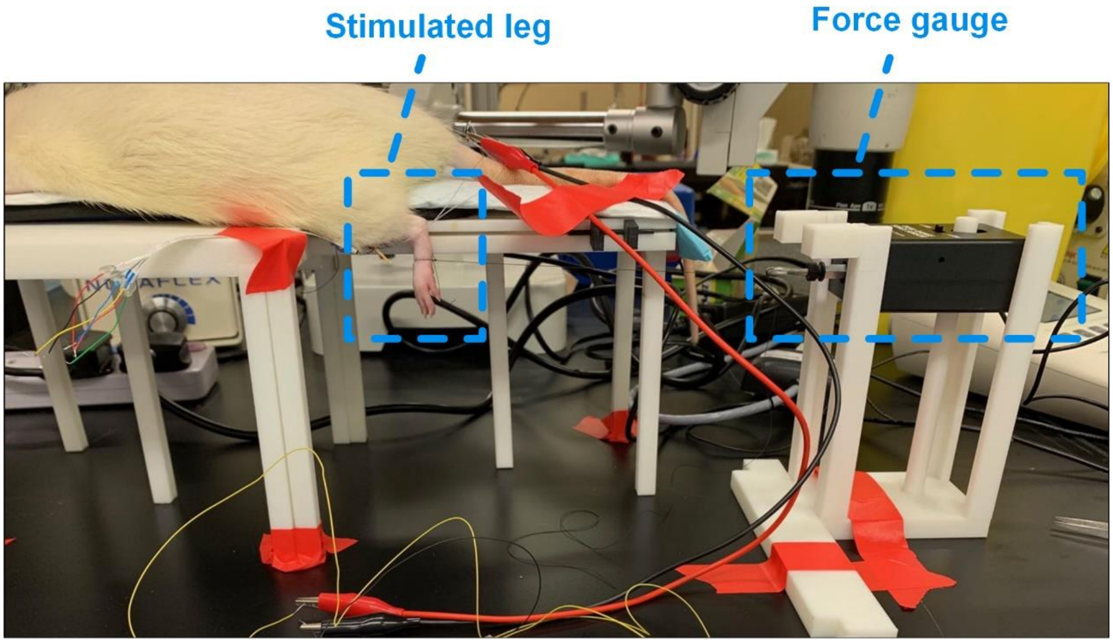

Figure S4. Testing set up for force measurement of the leg motion.

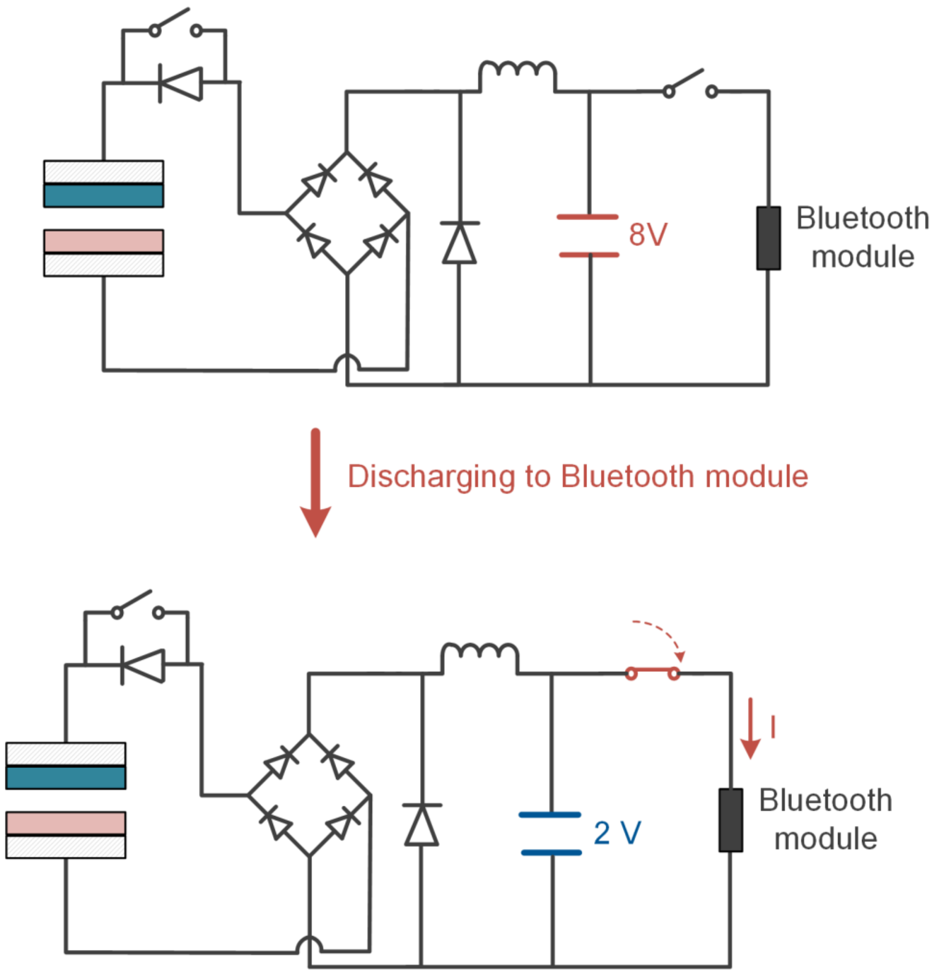

Figure S5. Schematic illustration showing the discharging to Bluetooth module. Upon connecting to the Bluetooth module, the capacitor discharges to it with its voltage dropping from 8 V to 2 V.

Supporting Videos

Video S1. Selective muscle stimulation with the D-T-TENG. The textile-based TENG is connected to the tibialis anterior muscle and the gastrocnemius muscle through two switches. Closing switch 1 leads the current to tibialis anterior muscle (highlighted in red in the video beginning), while the gastrocnemius muscle (highlighted in blue) is stimulated as switch 2 is closed. By simply varying the pressed switch, one TENG textile can stimulate two muscles effectively.

Video S2. Wearable textile-based communication board. The computer screen shows the corresponding icon to the pressed pixel.

Video S3. Wireless humidity and temperature sensing powered by D-T-TENG. The TENG textile is put in the front side of the shoe, and the textile-based switch locates at the rear end of the shoe. Charging circuit is attached to the side of the shoe. The Bluetooth module, as well as the switch for discharging is attached to the side of trousers. The smartphone is set to record the screen before moving. After 80 steps to make sure the charged-up voltage high enough for powering, the Bluetooth is powered by the capacitor through manually close the discharging switch. The screenshot showing the received data from the Bluetooth module is inserted as well.
